# Metabolite Profiling of Meridianin C In Vivo of Rat by UHPLC/Q-TOF MS

**DOI:** 10.1155/2021/1382421

**Published:** 2021-10-21

**Authors:** Guozhe Zhang, Linxia Xiao, Liang Qi

**Affiliations:** Department of Translational Medicine, Jiangsu Vocational College of Medicine, 283 South of Republic Road, Yancheng 224005, China

## Abstract

Meridianin C (MC), as a marine alkaloid, is a potent protein kinase inhibitor which exhibits good anticancer activity. However, the in vivo metabolism of MC has not been described to date. In this study, an ultra-high performance liquid chromatography/quadrupole time-of-flight mass spectrometry (UHPLC/Q-TOF MS) method is employed to investigate the in vivo metabolites of MC in rats. Plasma, bile, urine, and feces are collected after a single oral dose of MC. Protein precipitation, solid phase extraction (SPE), and ultrasonic extraction methods are used to prepare samples. Based on the mass spectral fragmentation patterns, elution order, and retrieving literatures, a total of 13 metabolites of MC were detected and tentatively identified, utilizing MetaboLynx software. The metabolic pathways of MC in rats include N- or O-glucuronidation, O-sulfation, N-hydroxylation, dihydroxylation, and trihydroxylation. The relative content of the metabolites in each kinds of biological samples is also evaluated. This study will help to understand the in vivo properties of MC for the future usage.

## 1. Introduction

It is universally acknowledged that the discovery and development of drugs is a time-consuming and costly procedure [1]. Absorption, distribution, metabolism, and excretion (ADME) of compound is one of the important considerations in the drug discovery process and can help investigate the drug after administration or injection. And so, ADME characteristics play an important role in drug design. However, drug discoveries and developments are commonly terminated in case of preclinical or clinical prodrug toxicity. Biotransformation of compound in vivo is thought to be one of the factors of drug toxicity [2]. Nowadays, the prodrug screening approach has been performed at a very early stage of drug discovery even drug development. Drugs after oral administration may always be biotransformed by intestinal flora and various metabolizing enzymes to different kinds of phase I and phase II metabolites, which are related to the parent drug. Furthermore, drug metabolic pathways are key factors in optimizing prodrugs for optimal pharmacokinetics properties. Metabolic pathways of prodrug can help finding latent active metabolites and minimizing potential safety due to biotransformation of reactive or toxic metabolites [3].

The LC/MS technology has been used as an important tool in drug discovery and drug development, owing to its selectivity, sensitivity, and speed. The quadrupole time-of-flight mass spectrometry systems can easily provide high-resolution mass and accurate mass data, which can efficiently help to calculate the molecular of metabolites. In the past few years, ultrahigh performance liquid chromatography (UHPLC) has become the common approach of ingredients separation for the LC/MS system in the cause of the drug metabolic study, for providing higher throughput, sensitivity, and/or improved selectivity than the traditional HPLC method. So, UHPLC combined with quadrupole time-of-flight mass spectrometry systems has become a commonly technology of the drug metabolism study [4].

**Figure 1:**
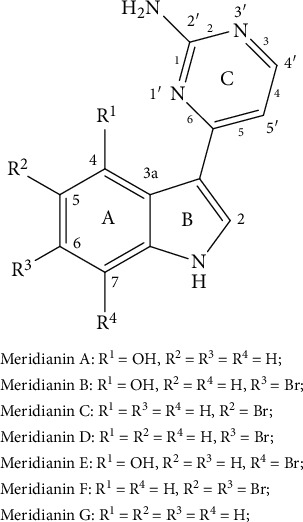
Structure, nomenclature of meridianins A–G.

Natural products have played close attention in drug discovery because of their easy degradation, environmental friendliness, and unique mechanisms of function [5–9]. Meridianins A–G (Figure 1) are natural products isolated from marine organisms. Since the isolation of meridianins A–E in 1998 [10], a total of seven of these alkaloids have been isolated (meridianins A–G) [11]. Meridianin alkaloids exhibit a lot of biological activities, such as anticancer [5, 10], protein kinase inhibitory activity [11], antimalarial activity [12, 13], antituberculosis activity [13], anti-Alzheimer's disease activity [14], and so on. Especially, meridianin C has been studied showing dramatical anticancer activities [15, 16]. To our knowledge, meridianins A–G are still not been used as drugs in clinical, and the in vivo metabolisms have not been studied yet. In the present study, UHPLC/Q-TOF MS is used to characterize and semiquantify the metabolites of MC in rat plasma, bile, urine, and feces for purpose of providing evidence of the course of meridianins in vivo.

## 2. Materials and Methods

### 2.1. Chemicals and Reagents

MC was prepared in our lab. The structure was determined using ^1^H NMR **(**Supplementary Figure S1**)** and mass spectrometry (MS) methods. The HPLC grade formic acid and acetonitrile were prepared form Thermo Fisher Scientific Co. Ultrapure water (18.2 MQ) was prepared with a Milli-Q water purification system (Millipore, France).

### 2.2. Animal Experiments [17]

Male Sprague-Dawley rats (220–250 g) were invited from the SPF (Beijing) Biotechnology Co., Ltd. (Beijing, China). They were adapted to new circumstances for one to seven days. The rats were fasted overnight before the drug metabolic study and only allowed free access to water.

Meridianin C powder was dispersed in 0.5% sodium carboxymethylcellulose (Na-CMC) solution at concentration of 10 mg/mL and then sonicated until a homogeneous suspension is obtained, which would be used for oral administration. Plasma samples were collected from the suborbital vein to obtain blank plasma. After oral administration of meridianin C at a dose of 100 mg/kg bodyweight to two rats, blood was collected from the suborbital vein at the time points of 0.5, 1, 2, 4, 8, 12, and 24 h, and all the blood samples were immediately centrifuged at 3,500 rpm for 10 min at 4°C to obtain supernatant plasma and stored at −80°C until analysis. Two rats were used for urine and feces sample collection. Both urine and feces were collected for 0–24 h after oral administration of MC at the dose of 100 mg/kg bodyweight. Urine samples were collected every two to four hours and stored at −20°C for later analysis. Feces samples were also stored at −20°C after drying. Two rats were used for bile collection in the period of 0–24 h. After collecting blank bile for a certain amount, rats were orally administrated with the same dosage of MC. The experimental protocol was approved by the Animal Experiment Ethics Review Committee of Jiangsu Vocational College of Medicine (LLSQ-2021-031106).

### 2.3. Preparation of Rat Biological Samples [17]

#### 2.3.1. Plasma

First, 200 *μ*L of mixed plasma was diluted with 600 *μ*L methanol, vortex-mixed for 2 min, centrifuged at 8,000 rpm for 10 min to precipitate proteins, and the supernatants were evaporated to dryness transferred in another tube. Second, the residue was reconstituted with 200 *μ*L methanol–water (80 : 20, v/v) and centrifuged at 14,000 rpm for 10 min. Finally, the supernatants were passed through a 0.22 *μ*m membrane filter, and 1 *μ*L sample was injected into the UHPLC/Q-TOF MS system for analysis.

#### 2.3.2. Urine

The solid phase extraction (SPE) method was used for handling urine samples with Bond Elut C18 cartridges (3 mL, 500 mg). Each cartridge was activated with 2 mL methanol and conditioned by 2 mL water. Then, 100 *μ*L of the spiked urine samples was loaded on to the cartridges. Then, 2 mL water and 2 mL methanol were added separately to the SPE cartridges to elute the matrix and the metabolites. The methanol elution was collected and dried. Then, the residue was redissolved with 200 *μ*L methanol–water (80 : 20, v/v) and centrifuged at 14,000 rpm for 10 min. After the supernatants were passed through a 0.22 *μ*m membrane filter, 1 *μ*L samples of the filtrates was injected into the UHPLC/Q-TOF MS system for analysis.

#### 2.3.3. Bile

Bile samples were also prepared with the SPE method, and the procedure was similar to urine sample, except that 100 *μ*L bile residue was redissolved with 150 *μ*L methanol–water (80 : 20, v/v).

#### 2.3.4. Feces

First, the spiked drying feces were grinded into fines. Weighted 1 g feces sample into a 50 mL tube and added 20 mL methanol–water (80 : 20, v/v). Second, 15 min ultrasonic extraction was used for feces sample preparation and then centrifuged at 3,500 rpm for 10 min to participate impurity. A curtain amount of the supernatants were continually centrifuged at 14,000 rpm for 10 min and passed through a 0.22 *μ*m membrane filter and used for mass analysis.

### 2.4. UHPLC and MS Conditions

The chromatography separation was carried out using a Waters ACQUITY BEH C18 column (2.1 × 100 mm, 1.7 *μ*m, Waters, Milford, MA, USA) at the column temperature of 40°C. The mobile phase consisted of (A) water containing 0.1% formic acid and (B) acetonitrile containing 0.1% formic acid with a gradient elution as follows: 0–1 min, 5% A; 1–10 min, 5%–95% A; and 10–12 min, 95% A. The flow rate was maintained at 0.4 mL/min, and 1.0 *μ*L of the control and MC-containing biological samples were injected into the system at each run.

Metabolite identification of MC in rat was performed on Waters quadrupole time-of-flight (Q-TOF) Xevo G2 (Waters, Manchester, UK) equipped with an electrospray ionization (ESI) source. The capillary voltage of the ESI source was set at 3.0 kV in the positive ionization mode. The cone voltage was set at 40 eV. Source and desolvation temperatures were set at 120°C and 450°C, respectively. The nebulization gas flow was set at 800 L/h. All data were acquired with the MS^E^ method in the resolution mode with 6 eV as low-collision energy and 20–60 eV ramp as high-collision energy. Both the molecular ions and the mass fragment ions could be acquired in a single run. Mass data were collected over the range of m/z 100–1500 in both functions. Leucine-enkephalin (m/z 556.2771) was used as real-time lock mass.

## 3. Results and Discussion

### 3.1. Mass Spectrometric Analysis of MC

**Figure 2:**
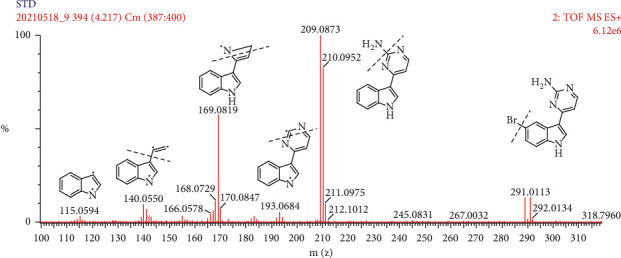
The product ion spectra and proposed fragmentation pathways of meridianin C.

The structures of meridianins A–G are shown in Figure 1. There are five main fragment ions of MC in the high-resolution spectrum (Figure 2). The speculated formula, observed and calculated mass, and mass errors are given in Table 1. The detected mass errors ranged from 0 to −5.3 mDa, indicating a good accuracy. The fragment ion structures are shown in Figure 2. Some fragments displayed a few ion clusters, and only one ion is given.

### 3.2. Metabolites Identification of MC [17]

A novel strategy was proposed for the systematic screening and characterization of MC metabolites. First, the MSE data of both the blank control samples and the biological samples containing MC were acquired using UHPLC-Q/TOF MS. Second, with the help of MetaboLynx^TM^ software, the molecular weights, the elemental compositions, and the proposed metabolic pathways of the metabolites derived from the accurate mass measurements can be automatically predicted. Finally, the structures of metabolites were justified based on the mass difference, the characteristic fragments of MC metabolites, and available literatures data.

The presence of the isotope pattern resulting from bromine atoms means that it is not lost via the metabolism. Both Br^79^ and Br^81^ isotopes were monitored by the mass spectrometer. The corresponding high-resolution mass spectrum displays identical isotopic cluster in an 1 : 1 ratio with two Da difference, thus confirming the presence of one bromine atom in the structure (such as m/z 289.01/291.01 in Figure 2). At the same time, the high-resolution mass spectrum without the isotopic cluster in 1 : 1 ratio indicates that the molecular has lost the bromine atom in the fragmentation process (such as m/z 209.08, 193.07, 169.08, and 140.05 in Figure 2).

**Figure 3:**
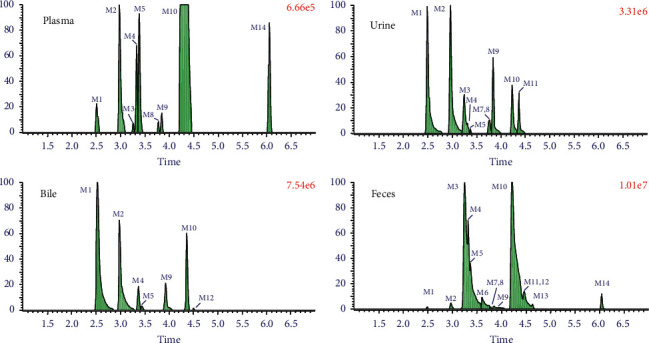
Extracted ion chromatograms (EICs) of the metabolites in meridianin C-containing rat biological samples identified by UHPLC/Q-TOF MS in the positive ion mode.

The extracted ion chromatograms (EICs) of the prototypes and metabolites in MC-containing rat biological samples are shown in Figure 3, which were generated with MetaboLynx software and also be confirmed by manually extracting in the matrix **(**Supplementary Figure S2**)**. The parameters of the 14 metabolites (including the prototype) are given in Table 2.

#### 3.2.1. Identification of Parent Drug in Rat Biological Samples (M10)

The metabolite M10 displayed the same molecular ion at 289.01/291.01 (C_12_H_9_BrN_4_, retention time 4.21 min), and the fragment ions were similar to those of MC. M10 was identified as the parent drug MC by comparing the retention time and accurate mass spectra with that of the authentic standard.

#### 3.2.2. Hydroxylated Metabolites (M3, M6, and M12)

M3, M6, and M12 displayed the same molecular ion at m/z 305.00 (C_12_H_9_BrN_4_O, retention times 3.24, 3.61 and 4.47 min), which was 16 Da (O) higher than the protonated molecule of MC. The characteristic fragment ions at m/z 289/291 indicate the loss of O atom. Therefore, M3, M6, and M12 were identified as hydroxylated metabolites of MC. By retrieving literature [18], the most probable conjugate cite was at the N position. The diagnostic ion of M3 and M6 at m/z 185 was 16 Da higher than the characteristic ion at m/z 169 of MC (Figure 2). Thus, it was proposed that the O atom conjugated at the A or B rings. 1-N-site in B ring was the most proposed conjugated site [19], and thereby, M3 with a relative higher amount should be 1-N-hydroxylate MC. M12, without the ion of m/z 185, was speculated to be 2′-N-hydroxylate MC.

#### 3.2.3. Hydroxylated and Sulfate Metabolites (M2 and M11)

**Figure 4:**
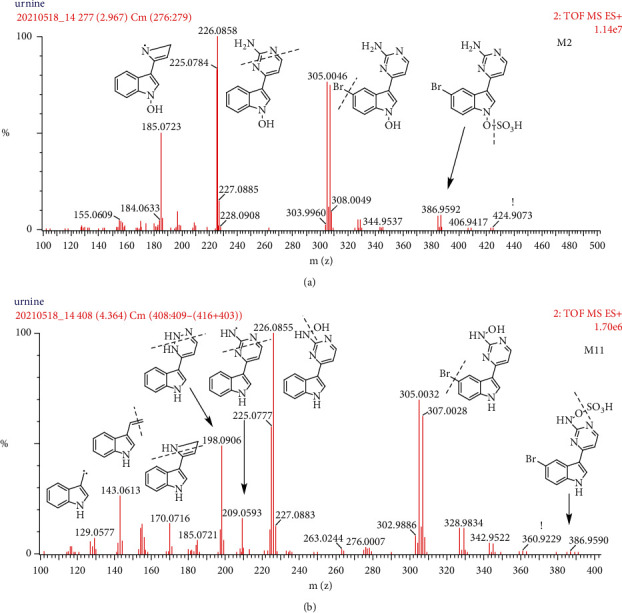
The product ion spectra and proposed fragmentation pathways of M2 and M11 in the positive ion mode.

M2 and M11 with protonated molecular isotopic ions at m/z 384.96 (C_12_H_9_BrN_4_O_4_S, retention time 2.97 and 4.37 min) were 80 Da (SO_3_) higher than the protonated molecule of M3. The characteristic fragment ions at m/z 331 (M + H−SO_3_) indicated that they were hydroxylated and sulfate metabolites of MC. As illustrated in Figure 4, the major fragment ions at m/z 305 [M + H−SO_3_]^+^, m/z 289 [M + H-SO_3_−O]^+^, m/z 226 [M + H−SO_3_−Br]^+^, and m/z 185 [M + H−SO_3_−Br−N_2_CH_3_]^+^ were found in the high-resolution spectrum. Both M2 and M11 contain the same fragment isotopic ion clusters of m/z 305/307, 289/291, and mono ion of m/z 209 in the high-resolution spectrum, which indicates the continuous loss of SO_3_, O, and Br by the accurate mass measurement. The diagnostic ion of M2 at m/z 185 was 16 Da higher than the characteristic ion at m/z 169 of MC (Figure 2), and thereby, the O atom conjugating at the A or B rings was put forward. 1-N-site in B ring was the most proposed conjugated site [18, 19]. While the absence of m/z 185 suggested that the O atom might conjugate at C ring, the most proposed conjugated site might be at 2′-N- in C ring. The subsequent fragmentation ions at m/z 170, 143, and so on are similar to those of MC, which was undergoing an easy loss of O atom followed by subsequent fragmentation. The characteristic fragment ion of M11 at m/z 198 generated a relatively high response, which was the absence of M2. It is tentatively deduced that the fragment m/z 198 was generated based on a rearrangement owing to fragmentation, when O atom conjugated at 2′-N position, as shown in Figure 1. Meanwhile, the 2′-N-O-S bond in C ring speculated easier break-up, encountering a high collision energy than 1-N-O-S bond in B ring because the extraction ion chromatograph of protonated molecular ion of M11 was hard to see. The calculated 1-octanol/water partition coefficient (CLog *P*) value was an important parameter for the metabolite identification, as an ingredient with a higher CLog *P* value may be eluted later when separated in the reversed-phase column. The CLog *P* values were calculated with ChemBioDraw Ver.14.0. The calculated CLog *P* values of 1-N and 2′-N-hydroxylated MC were 2.0 and 2.9, respectively. Thus, the hydroxylated and sulfate or glucuronide metabolites may present an early elution conjugating at 1-N-O- and a later elution at 2′-N-O-site. The high-resolution spectrum and the mass fragment of M2 and M11 are shown in Figure 4. M2 and M11 were tentatively identified as MC-1-N-O-sulfation and MC-2′-N-O-sulfation, respectively.

#### 3.2.4. Hydroxylated and Glucuronide Metabolites (M1 and M9)

**Figure 5:**
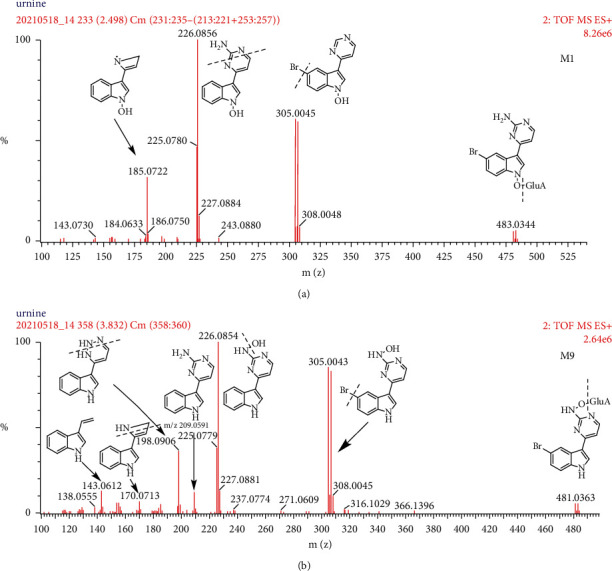
The product ion spectra and proposed fragmentation pathways of M1 and M9 in the positive ion mode.

M1 and M9 with protonated molecular ion at m/z 481.04 (C_18_H_17_BrN_4_O_7_, RT = 2.49 and 3.85 min) were 176 Da (C_6_H_8_O_6_) higher than the protonated molecular ion of hydroxylated MC. Glucuronide conjugates have their characteristic mass fragment in metabolite identification because the high collision energy spectrum of glucuronide conjugates always shows the loss of 176 Da (C_6_H_8_O_6_). As illustrated in Figure 5, the major fragment ions at m/z 305 [M + H−GluA]^+^, m/z 289 [M + H−GluA−O]^+^, and m/z 226 [M + H-GluA−O−Br]^+^ were found in the positive ion mode. M1 demonstrated the diagnostic ion at m/z 185, which was absent in M9. Repeating the same approach of hydroxylated and sulfate metabolite identification (Section 3.2.3), M1 and M9 were deduced to be MC-1-N-O-glucuronidation and MC-2′-N-O-glucuronidation, respectively. The proposed fragmentation pathways of M1 and M9 are shown in Figure 5.

#### 3.2.5. Glucuronide Metabolites (M4, M5, and M7)

These metabolites had the same protonated molecular ion at m/z 465.04 (C_18_H_17_BrN_4_O_6_, retention times 3.33, 3.38, and 3.75 min), which was 176 Da (C_6_H_8_O_6_) higher than the protonated molecular ion of MC. Diagnostic losses of 176 Da were indicating a glucuronide conjugate. In the high-resolution channel, they displayed the same fragment ion at m/z 289/291, and so, they were speculated to be glucuronide metabolites of MC. Among them, 1-N-O- and 2′-N-O- were the two possible conjugated sites [20–22]. We tentatively deduced M4 and M7 to be MC-1-N-glucuronidation and MC-2′-N-glucuronidation by their elution order.

#### 3.2.6. Dihydroxylated Metabolite (M8) and Trihydroxylated Metabolite (M13)

M8 demonstrates the protonated molecular ion at m/z 320.99 (C_12_H_9_BrN_4_O_2_, retention time 3.78 min), which was 32 Da higher than the protonated molecule of MC. The major fragment ions of M8 at m/z 305 [M + H−O]^+^, m/z 289 [M + H−2O]^+^, m/z 226 [M + H−O−Br]^+^, m/z 209 [M + H−2O−Br]+, and m/z 169 were found with high collision energy. M8 was identified as dihydroxylated metabolites of MC. M8 with two O atom conjugated at 1-N- and 2′-N*-*site, and 1,2′-N*-*OH-MC was the probable structure. Following similar strategy, M13 (m/z 336.99, C_12_H_9_BrN_4_O_3_, retention time 4.63 min) displayed the characteristic fragment ions at m/z 305 [M + H−2O]^+^, m/z 289 [M + H−3O]^+^, and m/z 226 [M + H−Br−3O]^+^, and so, M13 was identified as the trihydroxylated metabolite of MC.

#### 3.2.7. Other Metabolite (M14)

**Figure 6:**
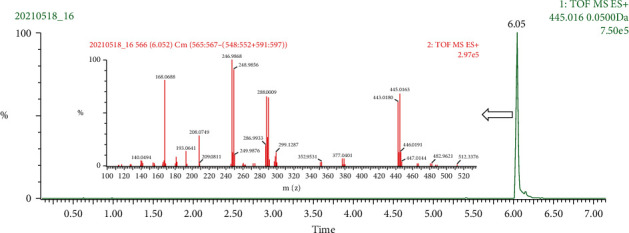
The EIC and high energy spectrum of M14.

M14 (m/z 445.016, C_18_H_13_BrN_4_O_5,_ retention time 6.05 min) was detected both in feces and plasma. The EIC and high energy spectrum of M14 are shown in Figure 6. The isotopic clusters at m/z 443.02/445.02, 288.00/290.00, and 246.99/248.99 in the ratio of 1 : 1 indicated that it was the metabolite of MC. However, MetaboLynx software gave a metabolism of (hydroxylation + glucuronide conjugation) + 2× alcohols dehydration, with the mass difference of 1.4 mDa. It is obvious that the skeleton of MC could not lose two H_2_O, and so, the alcohols dehydration may happen at the conjugated glucuronide site. Given this, further study is needed to justify this presumption.

### 3.3. Relative Content of Metabolites

The peak area was calculated using MetaboLynx (Figure 3). The content of each metabolite was displayed as the percentage of each extraction ion chromatograph peak areas relative to the total peak areas in plasma, urine, bile, and feces sample. As the biological samples at each time were spiked together in a certain period of time, the relative content could give some useful message for the ADME study of MC in the future. As given in Table 3, about 75.8% of MC was displayed as prototype in plasma, and 11.7%, 9.1%, and 57.7% of MC were displayed in bile, urine, and feces, respectively. About 51.5% hydroxylated and glucuronide metabolites, as well as 20.9% hydroxylated and sulfate metabolite, existed in bile. The relative amount of metabolites in urine was similar to those in bile. Fecal samples contained the most kinds of metabolites, and hydroxylated metabolites were the major phase I metabolites, occupying nearly 20% metabolites. 13.0% hydroxylated and sulfate metabolites were the major phase II metabolites.

### 3.4. Result of Analysis

The UHPLC/Q-TOF MS method, combined with MetaboLynx, was employed for rapid analysis of the metabolites of MC in vivo of rats. Acetonitrile and water containing 0.1% formic acid as the mobile phase and collision energy at 20–60 eV displayed good mass fragments of the metabolites. MetaboLynx can rapidly excavate the metabolites by comparing the blank control sample with the samples collected after administration of MC, which will dramatically reduce the mental work extraction ion chromatograph one by one and increase the accuracy and integrity. As the prototype contains bromine, we set the parameters of the isotopic mode, which will reduce the processing time from a few hours to a few minutes. This strategy can also be applied to evaluate the other compound metabolisms containing bromine or chlorine.

**Figure 7:**
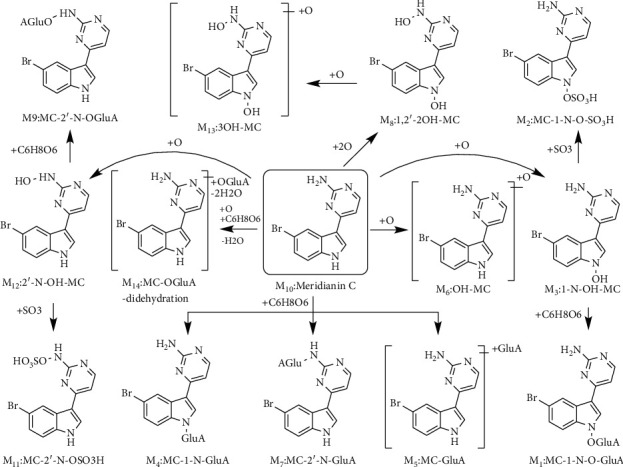
The proposed metabolic pathways of meridianin C in vivo of rat.

A total of 14 metabolites including the parent compound MC were found in vivo of rat. The proposed metabolic pathways of MC in rat are shown in Figure 7. The N- or O-glucuronidation, O-sulfation, N-hydroxylation, and dihydroxylation were the main metabolic pathways of MC. The extraction ion chromatograph of metabolites was found in samples, which was absent in blank controls. The high-resolution chromatograph exists in isotopic clusters at the ratio of 1 : 1, confirming the peaks are the metabolites of MC. MC-glucuronidation and MC-O-glucuronidation were the main metabolisms of MC in vivo comparing with MC-sulfation and MC-O-sulfation. The prototype of MC displayed a high concentration in plasma, bile, urine, and feces, proposing a higher bioavailability of MC after oral administration (Table 3). At the same time, natural products in vivo mainly undergo the phase II metabolism including glucuronide and sulfate conjugates to increase polarity and decrease toxicity, then excreting out of the body. The glucuronide or sulfate conjugation at MC detected the minor metabolism, compared with glucuronide or sulfate metabolites after hydroxylation of MC, which could boil down to no hydroxyl groups in the parent skeleton (Figure 1). It could be inferred that N-site was not active as O-site encountering the conjugated metabolism [21]. Furthermore, no sulfate conjugated at N-site metabolites was detected in all of the matrices, except a few N-glucuronide conjugation, so we inferred that the N-sulfate conjugation could hardly happen in vivo of rat [23]. The feces sample detected the most kinds of metabolites, and most of them also existed in other biological matrices. A relatively high amount of hydroxylated metabolites (almost 20%) of MC in feces suggests that MC was first transformed by intestinal bacteria, and then, MC and these phase I metabolites were absorbed into the blood, suffering further metabolism including N-, O-glucuronidation, and O-sulfation. Meanwhile, some phase II metabolisms including hydroxylation + sulfation (13.0%), glucuronide conjugation (7.6%), and hydroxylation + glucuronide conjugation (1.9%) were also detected in feces, which was quite different from those in the bile, suggesting that MC might undergo some first-pass metabolisms in the intestine after administration. However, this is contradicted to the relatively high bioavailability of MC in plasma. To our surprise, the amount of metabolites in plasma was quite different from those in urine and bile. The pharmacokinetics study of MC in plasma was needed in the future. A total of almost 88% phase II metabolites of MC were detected in bile. Thus, it can be inferred that MC also undergoes some first-pass metabolisms in the liver after absorption. The relative amount of metabolites in bile is similar to those in urine and different from those in feces. We tentatively speculated that in addition to hepatoenteric circulation, MC may encounter not only the phase I but also some phase II metabolisms happening in intestine with the metabolic enzymes and bacteria in the intestine, which catalyzes the drug's metabolic response and ultimately affects drug absorption.

## 4. Conclusions

In the present study, the UHPLC/Q-TOF MS method was used to explore the metabolites of MC in rat, after oral administration of MC, with the help of MetaboLynx software. A total of 14 metabolites were detected in rats and were tentatively identified based on the mass spectral fragmentation patterns, elution order, or retrieving literatures. N- or O-glucuronidation, O-sulfation, N-hydroxylation, dihydroxylation, and trihydroxylation were the major metabolisms of MC in vivo. Meanwhile, the relative content of each metabolite in each biological matrix was evaluated. Relatively high amount MC was displayed as prototype in plasma, proposing a high bioactivity of MC after oral administration. This study provides an experimental evidence for the metabolic process of MC and its family compounds in vivo and also provides a simple method for the rapid study of the metabolic process of bromine or chlorine containing compounds in vivo.

## Figures and Tables

**Table 1:** Empirical formula, observed and calculated mass, and mass errors of the principal fragment ions observed in the product ion mass spectrum of [MC + H]^+^.

No.	Predicted formula^a^	Calculated mass (Da)	Observed mass (Da)	Error (mDa)
1	C_12_H_9_N_4_Br^+^	289.0089	289.0131	−4.2
2	C_12_H_10_N_4_^+^	210.0905	210.0952	−4.7
3	C_12_H_7_N_3_^+^	193.0640	193.0684	−4.4
4	C_12_H_9_N_2_^+^	169.0766	169.0819	−5.3
5	C_10_H_9_N^+^	140.0500	140.0550	−5.0

^a^The predicted formula was regardless of the ions.

**Table 2:** UHPLC/Q-TOF MS analysis MC and its metabolites in rat plasma, bile, urine, and feces.

RT (min)	No.	Calculated mass	Observed mass (Da) fragment ions^a^	Error (mDa)^b^	Metabolite name	Formula	Matrix^c^
2.49	M1	481.0359	481.0365/483.03, 305.00/307.00, 226.09, 185.07	−0.6	Hydroxylation + glucuronide	C_18_H_17_BrN_4_O_7_	P, B, U, F
2.97	M2	384.9606	384.9613/386.96, 305.00/307.00, 226.09, 209.06, 197.08, 185.07	−0.7	Hydroxylation + sulfation	C_12_H_9_BrN_4_O_4_S	P, B, U, F
3.24	M3	305.0038	305.0118/307.01, 289.01, 226.09, 185.07	−8.0	Hydroxylation	C_12_H_9_BrN_4_O	P, U, F
3.33	M4	465.0410	465.0421/467.0406	−1.1	Glucuronide conjugation	C_18_H_17_BrN_4_O_6_	P, U, F
3.38	M5	465.0410	465.0416, 305.00/307.00, 226.08, 210.09, 185.07, 169.08	−0.6	Glucuronide conjugation	C_18_H_17_BrN_4_O_6_	P, B, U, F
3.61	M6	305.0038	305.0043/307.00, 289.01/291.01	−0.5	Hydroxylation	C_12_H_9_BrN_4_O	F
3.75	M7	465.0410	465.0408, 289.01/291.01, 210.09, 169.08	0.2	Glucuronide conjugation	C_18_H_17_BrN_4_O_6_	U, F
3.78	M8	320.9987	320.9985/322.9968	0.2	2x Hydroxylation	C_12_H_9_BrN_4_O_2_	P, U, F
3.85	M9	481.0359	481.0359/483.03, 305.00/307.00, 226.09, 209.06, 198.09, 143.06	0.0	Hydroxylation + glucuronide	C_18_H_17_BrN_4_O_7_	P, B, U, F
4.22	M10	289.0089	289.0089/291.01, 209.08, 169.08, 140.05	0.0	Parent	C_12_H_9_BrN_4_	P, B, U, F
4.37	M11	384.9606	384.9608/386.96, 305.00/307.00, 226.09, 209.06, 198.09, 185.07, 143.06	−0.2	Hydroxylation + sulfation	C_12_H_9_BrN_4_O_4_S	P, B, U, F
4.47	M12	305.0038	305.0041/307.00, 289.01/291.01, 169.08	−0.3	Hydroxylation	C_12_H_9_BrN_4_O	B, F
4.63	M13	336.9936	334.9955/336.9951, 318.97, 320.97	−1.9	3x hydroxylation	C_12_H_9_BrN_4_O_3_	F
6.06	M14	445.0148	443.0180/445.0161, 288.00/290.00, 246.99/248.99, 208.07, 168.07	−1.3	Hydration + glucuronide conjugation + 2× alcohols dehydration	C_18_H_13_BrN_4_O_5_	P, F

^a^Only characteristic fragment ions were shown. ^b^The mass error of molecular ions. ^c^U, urine samples; B, bile samples; P, plasma samples; F, fecal samples.

**Table 3:** Relative content of 14 metabolites of MC in rat plasma, bile, urine, and feces.

Metabolite	Metabolic pathway	Relative content (%)			
		Plasma	Bile	Urine	Feces
M1	Hydroxylation + glucuronide conjugation	1.4	45.5	29.2	0.3
M2	Hydroxylation + sulfation	6.5	28.2	29.7	1.0
M3	Hydroxylation	0.3	0.0	8.3	11.9
M4	Glucuronide conjugation	2.9	4.3	0.9	5.0
M5	Glucuronide conjugation	4.9	1.8	0.6	2.4
M6	Hydroxylation	0.0	0.0	0.0	4.2
M7	Glucuronide conjugation	0.0	0.0	1.5	0.2
M8	2x Hydroxylation	0.3	0.0	1.3	0.2
M9	Hydroxylation + glucuronide conjugation	0.6	6.0	12.5	1.6
M10	Parent	75.8	11.7	9.1	57.7
M11	Hydroxylation + sulfation	3.1	2.7	6.9	12.0
M12	Hydroxylation	0.0	0.3	0.0	3.1
M13	3x Hydroxylation	0.0	0.0	0.0	0.3
M14	Hydration + glucuronide conjugation + 2× alcohols dehydration	4.2	0.0	0.0	0.2

## Data Availability

The data used to support the findings of this study are included within the manuscript and are available from the corresponding author upon request.

## Supplementary Materials

Supplementary Materials Supplementary Figure S1. ^1^H NMR of meridianin C. Supplementary Figure S2. EIC of 14 metabolites.
