# Supplementary figures and images for: Metabolite Profiling of Meridianin C In Vivo of Rat by UHPLC/Q-TOF MS
Source: J Anal Methods Chem. 2021 Oct 21;2021:1382421. doi: 10.1155/2021/1382421 (PMC8553504; doi:10.1155/2021/1382421)

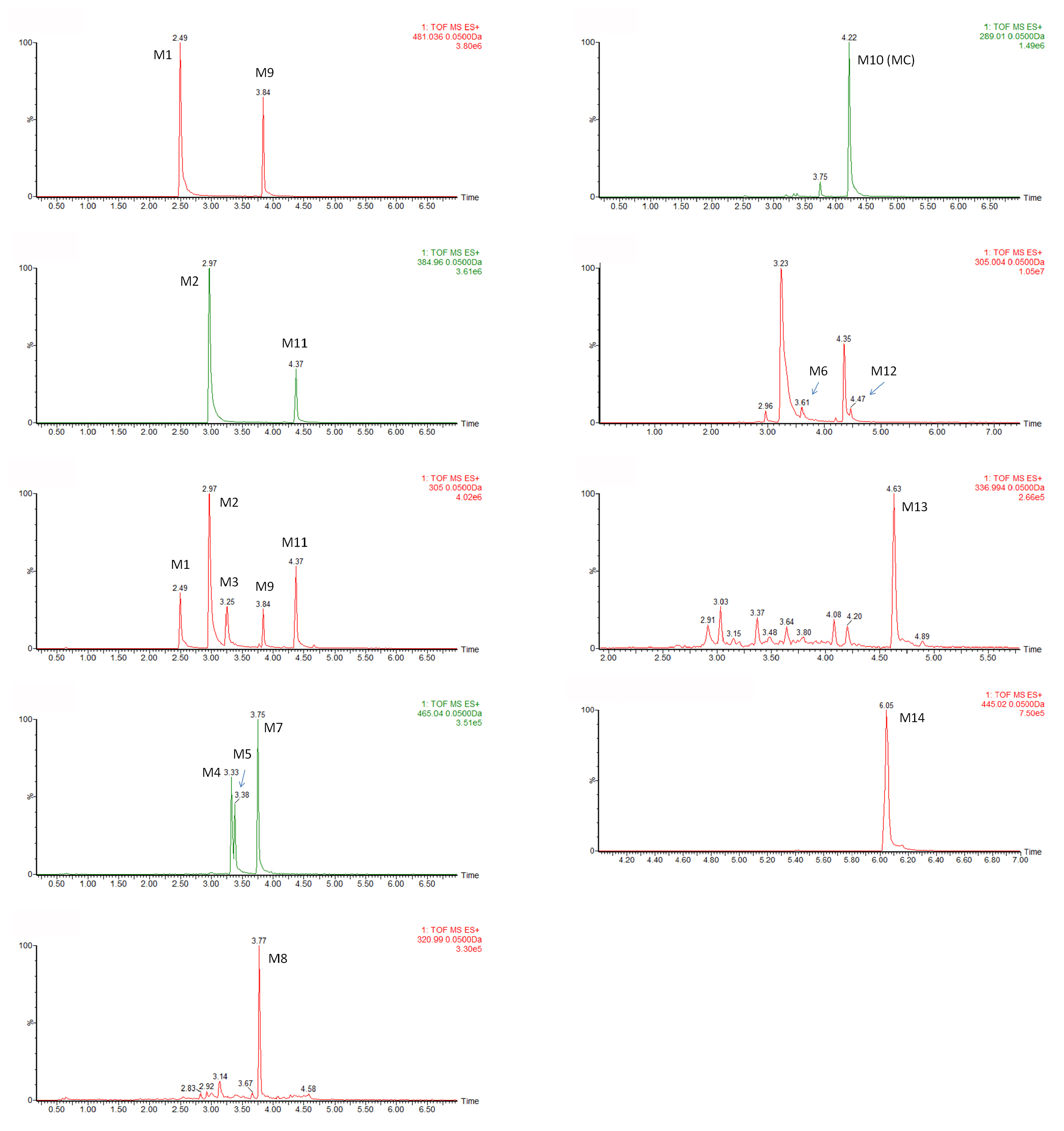

Supplement: Supplementary Materials — Supplementary Figure S1. 1H NMR of meridianin C. Supplementary Figure S2. EIC of 14 metabolites. [file 1382421.f1.png]
